# Bidirectional Association between Physical Activity and Dopamine Across Adulthood—A Systematic Review

**DOI:** 10.3390/brainsci11070829

**Published:** 2021-06-23

**Authors:** Adilson Marques, Priscila Marconcin, André O. Werneck, Gerson Ferrari, Élvio R. Gouveia, Matthias Kliegel, Miguel Peralta, Andreas Ihle

**Affiliations:** 1 CIPER, Faculty of Human Kinetics, University of Lisbon, 1499-002 Cruz Quebrada, Portugal; amarques@fmh.ulisboa.pt (A.M.); mperalta@fmh.ulisboa.pt (M.P.); 2 ISAMB, University of Lisbon, 1649-004 Lisbon, Portugal; 3 Faculty of Human Kinetics, University of Lisbon, 1649-004 Lisbon, Portugal; 4 Center for Epidemiological Research in Nutrition and Health, Department of Nutrition, School of Public Health, University of São Paulo (USP), São Paulo 05508-220, Brazil; andreowerneck@gmail.com; 5 Escuela de Ciencias de la Actividad Física, el Deporte y la Salud, Universidad de Santiago de Chile (USACH), Santiago 9170124, Chile; gerson.demoraes@usach.cl; 6 Departamento de Educação Física e Desporto, Universidade da Madeira, 9000-390 Funchal, Portugal; erubiog@uma.pt; 7 Interactive Technologies Institute, LARSyS, 9020-105 Funchal, Portugal; 8 Center for the Interdisciplinary Study of Gerontology and Vulnerability, University of Geneva, 1205 Geneva, Switzerland; matthias.kliegel@unige.ch (M.K.); andreas.ihle@unige.ch (A.I.); 9 Swiss National Centre of Competence in Research LIVES—Overcoming Vulnerability, Life Course Perspectives, 1015 Lausanne, Switzerland; 10 Department of Psychology, University of Geneva, 1205 Geneva, Switzerland

**Keywords:** neurotransmitter, brain, physical activity, mental health

## Abstract

Physical activity (PA) may influence the secretion of neurotransmitters and thereby have positive consequences for an individual’s vulnerability (i.e., reducing anxiety and depressive symptoms). This systematic review aims to analyse the potential bidirectional effects of exercise on dopamine from young adulthood to old age. The article search was conducted in PubMed, Scopus, and Web of Science in December 2020. The inclusion criteria were longitudinal and experimental study design; outcomes included dopamine and exercise; effect of exercise on dopamine and vice versa; adults; and articles published in English, Portuguese, or Spanish. Fifteen articles were included in the review. We observed robust findings concerning the potential effects of PA on dopamine, which notably seem to be observable across a wide range of participants characteristics (including age and sex), a variety of PA characteristics, and a broad set of methods to analyse dopamine. By contrast, regarding the potential effects of dopamine on PA, findings were mixed across studies. Thus, there are robust effects of physical exercise on dopamine. These findings further strengthen the idea that innovative approaches could include PA interventions for treating and preventing mental disorders. Therefore, it seems that PA is a potential alternative to deal with mental health issues.

## 1. Introduction

Dopamine is a monoamine neurotransmitter that is known to be modulated by physical activity (PA) and exercise [1]. The physiological roles of dopamine were first described in 1957 [2]. Dopamine is synthesized in both the central and peripheral nervous systems, acting as a signalling molecule. Dopamine has essential roles in regulating motor neurons [3], spatial memory function [4], motivation, and reinforcement learning [5]. In addition, dopamine plays a crucial role in maintaining chemical balance within the central nervous system [6]. Concerning vulnerability, an excess or lack of dopamine can cause mental disorders, such as depression [6,7].

Dopamine is critical for the motor system [8], and it has a well-established role in motor functioning [9,10]. Dopamine synthesis seems to affect the will to practice PA [11,12] and plays an important role in cognitive–motivational reward mechanisms for pursuing a certain behaviour such as PA [13]. This is because dopamine is a key neurotransmitter in the neural system, supporting cognitive control [14]. In turn, successful cognitive control is relevant for continuing PA engagement [15]. This mechanism is emphasized due to the association between the amount of striatal dopamine depletion and motor deficits observed in Parkinson’s disease [16].

On the other hand, PA seems to influence the central dopaminergic, noradrenergic, and serotonergic systems [17]. In this regard, it has been observed that an increase in PA seems to result in a corresponding increase in neurotransmitter activity [1,18,19,20]. PA is known to change the dopamine system in the central nervous system [1], increasing dopamine receptor availability [21]. This association may have positive consequences, such as reducing the severity of symptoms of anxiety, depression, and other mental-related issues [22,23,24]. Studies in patients with Parkinson’s disease also suggest that exercise may provide a preventive and non-pharmaceutical therapeutic approach [25,26,27]. However, the studies that examined the influence of PA on dopamine were mostly conducted in animals [28,29,30], and they either used PA as a stress model or compared exercise with other stressors [28].

The mechanism that increases dopaminergic activity during exercise is mostly related to fatigue [31,32,33]. A decrease in dopamine neurotransmission during PA would hasten the onset of fatigue, while an increase in dopamine neurotransmission might delay the onset of fatigue [31]. Studies that manipulated the increase of dopamine synthesis [34], stimulated extracellular dopamine release [35], inhibited dopamine reuptake [36], or directly activated dopamine neurons and/or dopamine receptors [37] were performed to better understand the role of dopamine in exercise-induced fatigue. Some of these manipulations have been successful in impacting exercise-induced fatigue.

These two different lines of research suggest that there is a bidirectional relationship between the practice of PA and dopamine [7,11,12,38]. Yet, so far, the underlying mechanisms are highly debated. Moreover, the existing empirical research investigating this reciprocal association showed high heterogeneity across multiple study parameters and provided mixed and, thus far, inconclusive results [39]. Therefore, to advance the understanding regarding the reciprocal relationship between PA and dopamine synthesis, we conducted a systematic review of the existing evidence concerning the potential PA and exercise effects on dopamine, and vice versa, across adulthood.

## 2. Materials and Methods

### 2.1. Search Strategy and Inclusion Criteria

The review was performed according to the guidelines of the Preferred Reporting Items for Systematic Reviews and Meta-Analyses [40]. We searched for articles that studied the relationship between PA and dopamine synthesis. As the relationship seems to be bidirectional [7,30,33,34], the research was extended to articles that explored the association in either direction. Thus, articles on the relationship between PA and dopamine, published in peer-reviewed journals up to 31 December 2020, were included.

The inclusion criteria of the articles were (a) longitudinal and experimental study design (study design criterion); (b) outcomes included dopamine and PA (outcome measure criterion); (c) effect of PA on dopamine and vice versa (relationship criterion); (d) adults (participants criterion); (e) articles published in English, Portuguese, or Spanish, regardless of the date of publication (language criterion). Studies were excluded from analysis if they were cross-sectional, did not have PA or dopamine synthesis as the outcome, and if participants were under 18 years of age.

In December 2020, a search was performed for articles in PubMed and Scopus databases and the meta-search engine Web of Science. The search was performed using the following terms: (“physical activity” OR exercise * OR train * OR sport *) AND (dopamine OR dihydroxyphenethylamine OR oxytyramine OR 3-hydroxytyramine) AND (“mental health” OR “mental disorder *” OR “mental problem *” OR “cognitive health” OR “cognitive disorder *” OR “cognitive problem *” OR depressi * OR anxiety OR dementia). Two authors screened titles and abstracts to identify articles that met the inclusion criteria. Two authors read the articles and decided whether they should be included in the analysis or excluded. The inclusion decision was consensual and in cases of disagreement, the decision was made by mutual agreement. A reverse search was conducted to screen publications that cited the identified studies. We also checked the reference lists of identified studies for further potential studies to include.

### 2.2. Data Extraction

From the articles, the author name(s), year of publication, study design, sample characteristics, country, method of the outcome variables, and main results were extracted. The extraction was carried out by one author, and coding was verified by two other authors.

### 2.3. Quality Assessment (Risk of Bias)

We used the Quality Assessment Tool for Quantitative Studies of the Effective Public Health Practice Project to assess the methodological quality and risk of bias of each study [41]. The study received a final rating as “strong”, “moderate”, or “weak”. The scale evaluated different aspects of the research: (A) selection bias, (B) study design, (C) confounders, (D) blinding, (E) data collection methods, (F) withdrawals and dropouts, (G) intervention integrity, and (H) analysis. Each item received a final score of “strong”, “moderate”, or “weak”, according to the recommendations of the scale. Each study received a final rating as “strong” (when it received no “weak” ratings), “moderate” (when the study received one “weak” rating), or “weak” (two or more “weak” ratings). As we included clinical trials and cohort studies, we did not consider the item of selection bias for the clinical trials, and we did not consider the items of blinding and intervention integrity for cohort studies. The quality assessment is presented in Table 1. The majority of the papers were classified as “weak”, indicating a considerable risk of bias. There were only two studies classified as “moderate” [42,43], and one classified as “strong” [44]. The lack of adjustment for potential confounders in the analyses and the lack of blinding were the major sources of bias in the articles.

### 2.4. Synthesis of Results

Among the articles, there was great heterogeneity in the different parameters analysed. This made it impossible to carry out a meta-analysis. To facilitate the interpretation of the results, the data of each article were collected and presented consistently. As the articles presented relationships in different directions, a table was created for articles that analysed the effect of PA on dopamine, and another table was created for articles that analysed the effect of dopamine on PA.

## 3. Results

### 3.1. Literature Search

**Figure 1:**
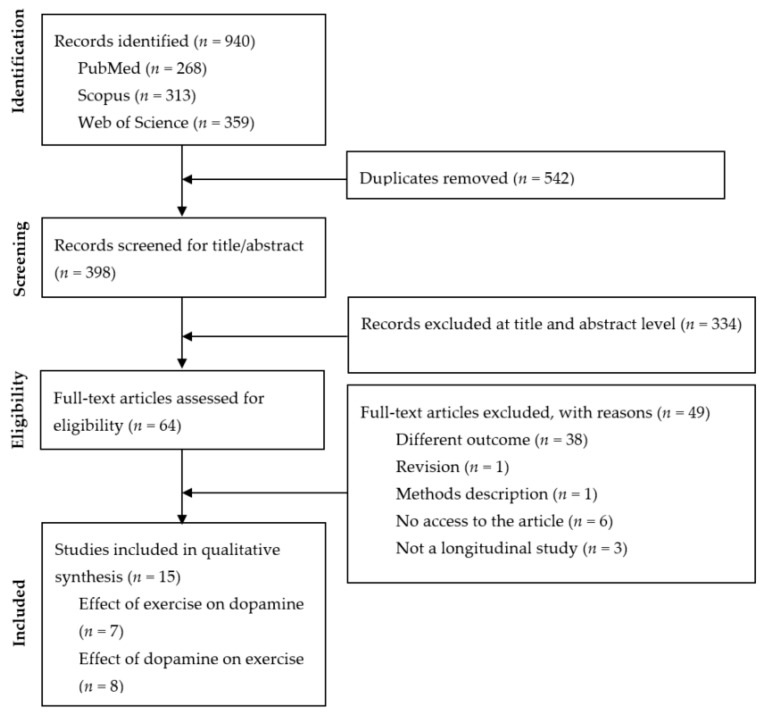
Flow diagram of study selection.

From the database search, 940 records were identified. Among those 940 records, 542 were duplicates and were eliminated. The remaining 398 records were analysed based on the title and abstract for potential inclusion in the subsequent full read. At this stage, 334 records were eliminated. The full text of 64 records was evaluated, and 47 were excluded for the following reasons: different outcomes (*n* = 38), the article was inaccessible (*n* = 6), the study design was not experimental or longitudinal (*n* = 3), the article was classified as a method description article (*n* = 1), or the article was a review (*n* = 1). Therefore, 15 articles were included in the systematic review. From the 15 included studies, 7 investigated the effect of PA on dopamine, while the other 8 examined the effect of dopamine on PA. The flow diagram of the study search is presented in Figure 1.

### 3.2. Effect of Exercise on Dopamine

The characteristics and main findings of the seven studies analysing the effect of PA (exposure) on dopamine (outcome) are presented in Table 2.

#### 3.2.1. Participants Characteristics

The number of participants in the seven included studies ranged from 6 [45,47] to 20 [42]. Regarding sex, five studies had a mixed (men and women) sample [42,45,48,49,50], while two studies had a sample only including men [46,47]. Concerning age, in four studies, the sample was composed of young adults (mean age ranged from 18 to 30 years across studies) [45,46,47,50]. In the other three studies, the sample was composed of middle-aged and/or older adults (mean age ranged from 51.7 ± 2.3 to 66.7 ± 5.9 years across studies) [42,49]. Finally, four studies were performed in special clinical popula-tions, one among hypertensive individuals [48] two among individuals with Parkin-son’s disease [42,49], and the last one among individuals addicted to methampheta-mine [50].

#### 3.2.2. Exercise Training Protocols and Interventions

Each exercise training protocol and intervention was unique among the seven in-cluded studies. Participants in three studies performed exercise interventions: partici-pants in one study performed bicycle ergometer exercises three times a week for a total of 10 weeks [48], participants in another performed 60-min sessions of individualized resistance training three times a week for a total of eight weeks [50], and participants in the most recent study performed 40–60-min sessions of aerobic cycling three times a week for a total of eight weeks [42]. Three studies used maximal and submaximal exer-cise training protocols, including a two-step test [45], a combined supine followed by a cycle ergometer test [46], and a forearm with hand ergometer test [47]. In the remain-ing study, individuals with Parkinson’s disease performed a right-foot sequential ex-tension/flexion protocol at each participant’s pace [49].

#### 3.2.3. Outcome Measures

To examine dopamine, three studies collected blood samples, of which two inves-tigated dopamine-ß-hydroxylase [45,46], and one analysed catecholamine (adrenaline, noradrenaline, and dopamine) [47]. Two studies used tomography scans; one per-formed scans during exercise on the dopaminergic system [49] and the other one (^11^C) used raclopride positron emission tomography scans to determine the effect of aerobic exercise on the repetitive transcranial magnetic stimulation-evoked release of endog-enous dopamine in the dorsal striatum [42]. One study collected urine samples [48]. Lastly, one study used a radioligand technique to determine dopamine D2/D3 receptor availability using (^18^F) fallypride [50].

#### 3.2.4. Main Findings

Overall, six studies found a positive effect of physical exercise on dopamine and one study found no effect. Regarding the exercise intervention studies, one study showed that urine dopamine significantly increased at four weeks [48], another pre-sented an increase in striatal dopamine D2/D3 receptor availability but no changes in the extrastriatal regions [50], and one found an increased repetitive transcranial mag-netic stimulation-evoked dopamine release in the caudate nucleus [42]. Among the remaining four studies, two found that exercise increased blood plasma dopamine [45,46], and the others observed a significant dopamine release in the ventromedial striatum and a lack of dopamine release in the putamen during exercise [49]. However, one study did not observe significant changes in dopamine concentration after exercise [47].

### 3.3. Effect of Dopamine on PA

The characteristics and main findings of the eight studies analysing the effect of dopamine (exposure) on PA (outcome) are presented in Table 3.

#### 3.3.1. Participant Characteristics

Among the eight included studies, the number of participants ranged from 5 [52] to 1635 [44]. In four studies, the sample was exclusively composed of men [43,52,54,55], while the remaining six studies had samples composed of both men and women [51,56]. Concerning age, most studies were with young adults (mean range between 22 and 26 years). In three studies, participants had a mean age between 59 and 78 years [44,51], and one study did not reveal participants’ ages [52]. Of the eight studies, four studies were performed with special populations: two in athletes [54,56]; one among individuals with severe chronic congestive heart failure [51]; and one in sedentary individuals at risk of disability [44].

#### 3.3.2. Dopamine-Related Exposure

Seven studies used drug exposure, while one study analysed dopamine without exposure to drugs. Regarding the studies that used drug exposure, in three studies, dopamine was directly administered to the participants at different doses, 2 µg/kg/min [51] and 3 µg/kg/min [43,52]. The other four studies administered dopamine inhibitor drugs, including, metoclopramide (20 mg) [55], domperidone (30 mg) [53], bupropion (2 × 300 mg) [54], methylphenidate (40 mg) and reboxetine (8 mg) [56]. The study without exposure to drugs analysed single nucleotide polymorphisms of dopamine-related genes [44].

#### 3.3.3. Outcome Measures

The seven studies that used drug exposure also assessed PA by performing submaximal or maximal cycle exercise protocols [43,51,52,53,54,55,56]. The remaining study assessed habitual PA by accelerometry [44].

#### 3.3.4. Main Findings

Concerning the three studies that directly administered dopamine, no significant effects were observed in the cardiac system or aerobic exercise capacity [43,51,52]. The studies that administrated dopamine inhibitor drugs found contradicting results. On the one hand, two studies observed that the dopamine inhibitor drugs had a positive effect on performance [54] and fatigue-related decrements in the peak velocity of pro-saccades [56]. On the other hand, one study showed that blocking dopamine receptors appeared to be detrimental to exercise performance [55]. In another study, it was found that dopamine D2-receptors were not involved in the hypoxia-induced decrease at the maximal heart rate [53]. The study that did not administer any drugs suggested a positive association between the expression of dopamine-related genes and habitual PA [44].

## 4. Discussion

We systematically reviewed the existing research on reviewed the existing re-search on the bidirectional relationship between exercise or PA and dopamine. Regarding the effects of exercise on dopamine, the majority of included studies (6 out of 7) reported potential effects of exercise on dopamine [42,45,46,48,50]. Exercise resulted in increased urine dopamine [48], increased striatal dopamine D2/D3 receptor availability [50], increased dopamine release in the caudate nucleus [42], dopamine release in the ventromedial striatum [26], and increased blood plasma dopamine [45,46]. Only one study did not observe significant changes in dopamine concentration after exercise [47]. Findings of the potential effects of dopamine on PA were mixed across studies. No significant effect was observed on the cardiac system or aerobic exercise capacity in three studies that administered dopamine [43,51,52]. Contradictory results were found in studies that administrated dopamine inhibitor drugs, with dopamine inhibitor drugs, having a positive effect on perfor-mance [54] and fatigue-related decrements in the peak velocity of prosaccades [56]. Nonetheless, blocking dopamine receptors seems to be detrimental to exercise performance [55], and a significant positive association between the expression of dopa-mine-related genes and habitual PA was observed [44].

The mental health benefits of PA are well documented [57]. Regular PA and exercise contribute to improved mental health through improving cognition [58,59,60], increasing BDNF levels in the brain [61], and brain plasticity [59]. Furthermore, people who are more physically active and have better fitness levels are less likely to have mental illnesses such as depression and anxiety [62,63,64]. From the results of this systematic review, it is possible to observe that exercise has a positive and significant effect on dopamine synthesis. It was observed that six out of seven studies reported effects of exercise on dopamine [42,45,46,48,50], which means that exercise can have a positive impact on mental health. For this reason, PA and exercise are, in some cases, effective behaviours for the prevention of mental illness and have a positive effect on the treatment of mental health problems [57]. Therefore, treatment guidelines for mental health should emphasize exercise [65]. To determine how exercise can benefit mental health, it is important to study its link to chemicals associated with stress and mental health [22,66]. One theory is that PA and exercise trigger the release of dopamine, which can improve mood [60]. It is important to emphasize that dopamine is a monoamine that regulates, and experiences regulatory influence from, the other two major monoamine neurotransmitters, noradrenaline and serotonin. This means that the monoamine system mediates the exercise-induced improvement of various brain functions [60].

As exercise is a complex behaviour (i.e., its frequency, duration, intensity, context, and other variables associated with its performance have to be considered), there could be different results among the analysed studies because studies differed substantially regarding the characteristics of the respective exercise. However, the majority of the studies spoke for the possibility that the relationship of exercise influencing dopamine may be observable across a variety of exercise characteristics [42,45,46,48,49,50]. These results reinforce the idea that the importance is more in the behaviour itself than in the way the PA behaviour is performed. For curing mental health issues, of which low dopamine synthesis is one of the main causes [6,7], these results are encouraging because innovative perspectives could include the prescription of exercise as a treatment.

It is well known that dopamine has a role in motor functioning [9,10], which may influence a willingness for PA [11,12]. Studies with animals have shown that blocking dopamine receptors results in less engagement in exercise voluntarily [67]. Thus, it can be speculated that in humans a central component might control PA levels as part of a biological regulation scheme, such as dopaminergic function. This hypothesis is based on the fact that the dopaminergic system has implications in many brain functions, including: rewards, motivation, learning, stimuli response, and movement [68]. As a result, it is suggested that dopaminergic signalling acts in a dependent and independent way in the regulation of PA. Nonetheless, findings of the potential effects of dopamine on PA were unclear. No significant effect was observed on the cardiac system or aerobic exercise capacity in three studies that administered dopamine [43,51,52]. Contradictory results were found in the studies that administrated dopamine inhibitor drugs, with dopamine inhibitor drugs having a positive effect on performance [54] and fatigue-related decrements in the peak velocity of prosaccades [56]. Blocking dopamine receptors in one study seemed to be detrimental for physical exercise performance [55], while in another study it was found that dopamine D2-receptors were not involved in the hypoxia-induced decrease in the maximal heart rate [53]. A significant positive association between the expression of dopamine-related genes and habitual PA was observed [44]. Thus, only the study examining the link between dopamine without exposure to drugs and real-life PA was able to demonstrate a coherent positive influence of dopamine on PA, regardless of the differences in participants’ characteristics. One may argue that the non-significant studies may have a relatively small sample [43,51,52,53], but they were comparable in size to other studies observing significant relationships [54,55,56]. Thus, from these results, it is not possible to determine that there is a relationship. More studies are needed to clarify whether the levels of dopamine in the brain influence the willingness to practice PA and, consequently, the levels of PA.

It is important to mention methodological details that systematically varied across the eight studies that analysed the effect of dopamine on PA. The seven studies that used drug exposure assessed PA by performing submaximal or maximal cycle exercise protocols and reported either non-significant or mixed results [43,51,52,53,54,55,56]. Notably, the remaining study, which analysed dopamine without exposure to drugs, assessed habitual PA using accelerometers [44]. This suggests that the particular PA assessment may constitute an important methodological factor for the potential effects of dopamine on PA. Similarly, the findings suggest that laboratory drug administration can differ from neutral conditions among people with different gene expression. Therefore, it is plausible to infer that acute dopamine release may not be determinant for exercise performance, but a constantly greater release of dopamine through determined genetic expressions may be associated with higher levels of habitual PA in the long term. However, more studies are still needed to investigate the association in neutral conditions.

The current systematic review findings should be interpreted in light of some limitations. Although study quality was assessed, studies were not weighted or ranked, nor were any removed from the review. Therefore, studies with weaker quality were given no less importance than findings from studies with greater quality. Nevertheless, most studies on the potential effects of dopamine on PA presented a ‘weak’ quality assessment. Furthermore, the studies included presented a variety of methods to assess both PA and dopamine, leading to difficulties and caution in the comparisons presented. Grey literature was not searched or included, and six studies were inaccessible. In addition, there was a lack of trials, indicating that larger representative trials are still warranted. Moreover, we found few studies investigating the effect of PA on dopamine and no studies investigating the effect of dopamine on PA in the long term, which also warrants further investigation. Finally, the present review did not focus on the different aspects of the dopamine system. Future research will have to focus on this issue and determine the detailed mechanisms of PA-related changes in the different roles of dopamine in the system.

## 5. Conclusions

Summarizing the prior empirical evidence on the reciprocal relationship between PA and dopamine, we observed robust findings concerning potential effects of PA on dopamine, which seem to be observable across a wide range of participant characteristics, a variety of PA characteristics, and a broad set of methods to analyse dopamine. By contrast, for potential effects of dopamine on PA, findings were mixed across studies, with only the study of neutral conditions (i.e., examining dopamine without exposure to drugs) able to demonstrate a coherent positive effect of dopamine on habitual PA measured by accelerometers.

With respect to translating this research to clinical practice, it is appropriate to suggest that health professionals should encourage engagement in PA as a strategy to improve dopamine levels and possibly promote mental health. Furthermore, these findings reinforce the chain of thought suggesting that PA should be considered as an intervention strategy for improving mental health.

## Figures and Tables

**Table 1:** Quality assessment.

	Items								
Source	Selection Bias	Study Design	Confounders	Blinding	Data Collection Methods	Withdrawals and Drop-outs	Intervention Integrity	Analysis	Total
Effect of Exercise on Dopamine									
Wooten & Cardon [45]	-	Strong	Weak	Weak	Strong	Strong	Strong	Strong	Weak
Péronet et al. [46]	-	Strong	Weak	Weak	Strong	Strong	Strong	Strong	Weak
Hartling et al. [47]	-	Strong	Weak	Weak	Strong	Strong	Strong	Strong	Weak
Kinoshita et al. [48]	-	Strong	Weak	Weak	Strong	Strong	Strong	Strong	Weak
Nozaki et al. [49]	-	Moderate	Weak	-	Strong	Strong	Strong	Strong	Weak
Robertson et al. [50]	-	Strong	Weak	Weak	Strong	Moderate	Strong	Strong	Weak
Sacheli et al. [42]	-	Strong	Weak	Strong	Strong	Strong	Strong	Strong	Moderate
Effect of Dopamine on Exercise									
Maskin et al. [51]	-	Strong	Weak	Weak	Strong	Strong	Strong	Strong	Weak
Boetger & Ward [52]	-	Strong	Weak	Weak	Strong	Strong	Strong	Strong	Weak
Lundby et al. [53]	-	Strong	Weak	Weak	Strong	Strong	Strong	Strong	Weak
Watson et al. [54]	-	Strong	Weak	Weak	Strong	Strong	Strong	Strong	Weak
Janssen et al. [43]	-	Strong	Weak	Moderate	Strong	Strong	Strong	Strong	Moderate
Tedjasaputra et al. [55]	-	Strong	Weak	Weak	Strong	Strong	Strong	Strong	Weak
Connell et al. [56]	-	Strong	Weak	Weak	Strong	Strong	Strong	Strong	Weak
Rosso et al. [44]	Moderate	Moderate	Strong	-	Strong	Moderate	Strong	Strong	Strong

**Table 2:** Characteristics and the main results of studies analysing the effect of exercise on dopamine.

Source	Study Design, Sample Characteristics (n, Sex, Age in Years), Country	Outcome Measures (Dopamine)	Exercise Training Protocol or Interventions	Main Findings
Wooten & Cardon [45]	Experimental study, 6 participants (3 men, 3 women), aged 18 to 20, USA.	Blood sample measurements of DBH activity.	The cold pressor test involved immersion of the hand in ice water for 3 min. The exercise was a two-step test (21-cm steps) performed at the fastest tolerable rate.	Cold pressor test and exercise resulted in small but significant elevations of plasma DBH activity. No significant change occurred during tilting.
Péronet et al. [46]	Experimental study, 7 football players, male, mean age 19 ± 1.0, Canada.	Blood samples were taken during the last min of each condition for measurements of DBH activity.	Supine test for 20 min, 3 min of handgrip exercise, and 10 min in a standing position at end of a supramaximal cycle ergometer test.	DBH activity increased above resting level during supramaximal dynamic exercise.
Hartling et al. [47]	Experimental study, 6 males, mean age 25.0 ± 4.0, Denmark.	Catecholamines were collected from the brachial artery and the deep vein.	Dynamic forearm exercise, rate of 50 contractions/min on a spring-loaded hand ergometer. Three min bouts of exercise were performed with 15 min intervals until complete exhaustion.	Adrenaline and noradrenaline increased. Dopamine concentrations did not change.
Kinoshita et al. [48]	Experimental study, 12 hypertension patients (4 men, 8 women), mean age 51.7 ± 2.3, Japan.	24-h urine and fasting blood samples were collected at weeks 0, 1, 2, 4, 7, and 10 of exercise.	Bicycle ergometer exercises, 3 times per week for 10 weeks.	Urine dopamine increased significantly in the 4th week, from 386 ± 9.4 µg/day at week 0 to 524 ± 6.3 µg/day.
Nozaki et al. [49]	Retrospective observational study, 12 Parkinson’s disease patients (6 men, 6 women), mean age 64.9 ± 7.8, Japan.	Tomography scans during right-foot movement in DBS-off and DBS-on conditions.	Right-foot sequential extension/flexion movements at participant’s own pace (close to 0.5 Hz).	Lack of dopamine release in the putamen and significant dopamine release in the ventromedial striatum by STN-DBS during exercise.
Robertson et al. [50]	Retrospective observational study, 19 methamphetamine-dependent participants (11 men, 8 women), mean age 29.8 ± 5.9, USA.	D2/D3 BPND was determined using (^18^F) Fallypride.	Exercise training group (EX): 1 h individualized exercise sessions (resistance training) 3 days/week for 8 weeks. Education control group: health education sessions, 1 h, 3 times/week for 8 weeks.	EX showed a significant increase in striatal D2/D3 BPND, no changes in D2/D3 BPND in extrastriatal regions.
Sacheli et al. [42]	RCT, 20 participants with mild to moderate (Hoehn & Yahr stages I–III) idiopathic Parkinson’s disease, (13 men, 7 women), mean age 66.7 ± 5.9, Canada.	(^11^C) raclopride positron emission tomography scans to determine the effect of aerobic exercise on the repetitive transcranial magnetic stimulation-evoked release of endogenous dopamine in the dorsal striatum.	Aerobic exercise: 40–60 min of cycling. Control: series of seated and standing stretches and low-impact exercises. Both: 3 times per week for 3 months (36 sessions).	The aerobic group demonstrated increased repetitive transcranial magnetic stimulation-evoked dopamine release in the caudate nucleus.

Abbreviations: D2/D3 BPND, dopamine D2/D3 receptor availability; DAT, dopamine transporter availability; DBH, dopamine-ß- hydroxylase; DBS, deep brain stimulation; RCT, randomized controlled trial; STN, subthalamic nucleus.

**Table 3:** Characteristics and the main results of studies analysing the effect of dopamine on physical activity and exercise.

Source	Study Design, Sample Characteristics (n, Sex, Age in Years), Country	Outcome Measures (PA)	Dopamine-Related Exposure	Main Findings
Maskin et al. [51]	Experimental study, 13 patients (9 men, 4 women) with severe chronic congestive heart failure, mean age 59 (range 48–72), USA.	Bicycle ergometer. The initial workload was 25 W for 3 min, and this load was increased every 3 min by 12.5 W until exhaustion.	Drug exposure: Dopamine was infused at an initial rate of 2 µg/kg/min for 15 min	DA exerted a slight chronotropic effect but did not improve ventricular performance during maximal exercise.
Boetger & Ward [52]	Controlled trial, 5 healthy males, USA.	A series of square-wave sub-anaerobic work-rate step tests on a bicycle ergometer was administered to each participant on 2 days.	Drug exposure: 3 µg/kg/min of dopamine at least 10 min before the dopamine test to ensure equilibration	Steady-state VE, VCO_2_. And VO_2_ were unchanged by dopamine infusion, both during unloaded pedalling and at the heavier workload.
Lundby et al. [53]	RCT, 12 sea-level natives (5 women, 7 men), aged 26 ± 1.4, Denmark, Switzerland, and Italy.	Two consecutive maximal exercise bouts, separated by an interval of 1 h, were performed on 4 separate occasions: at sea level and on day 1 (HA1, 24 h after arrival), day 3 (HA3), and day 5 (HA5) at high altitude. Five-minute warm-up at 120 W on a Monark 848 cycle ergometer and maximal exercise test. The protocol was designed to exhaust the participants within 3–5 min.	Drug exposure: 30 mg of domperidone (orally)	Hypoxic exercise in humans activated D2-receptors, resulting in a decrease in circulating levels of noradrenaline. However, dopamine D2-receptors were not involved in the hypoxia-induced decrease at the maximal heart rate.
Watson et al. [54]	Experimental randomized double-blind study, 9 healthy males, cyclists or triathletes, aged 22.7 ± 4.3, Belgium.	Constant cycle exercise for 60 min at a workload corresponding to 55% W_max_, followed by a TT to measure performance. The TT required the participants to complete a predetermined amount of work equal to 30 min at 75% W_max_ as quickly as possible.	Drug exposure: Placebo or 2 × 300 mg bupropion	Performance in warm conditions is enhanced by acute administration of a dual dopamine/noradrenaline reuptake inhibitor.
Janssen et al. [43]	Prospective placebo-controlled randomized study, 13 healthy males, aged 23 ± 3, Belgium.	Each participant underwent a physician-supervised standard incremental CPET until the symptom-limited maximum. The work rate was increased by 30 W per minute after 1 min pedalling at 0 W.	Drug exposure: Dopamine (3 µg/min/kg) or placebo infusion (0.9% NaCl)	Inhibition of peripheral chemoreflex function with dopamine decreased the V_E_/V_CO2_ slope during dynamic exercise, with no change in aerobic exercise capacity.
Tedjasaputra et al. [55]	Experimental study with placebo control, 12 healthy males, aged 25 ± 6, Canada.	Two incremental staged cycling exercise sessions. The initial power output was set to 50 W, and the power output was increased by 25 W every 2 min until the ventilatory threshold was reached.	Drug exposure: Placebo or a DA receptor blocker (metoclopramide 20 mg)	DA blockade did not change O_2_ consumption, CO_2_ production, or respiratory exchange ratio at different exercise intensities. DA blockade decreased maximal cardiac output, VO2_max_, and TTE. Blocking DA receptors appeared to be detrimental to exercise performance.
Connell et al. [56]	Double-blind, placebo-controlled, repeated-measures randomized crossover study, 12 trained cyclists (7 women, 5 men), mean age 25 (19–45), New Zealand.	Three experimental trials involving 180 min of continuous cycling at a work rate equivalent to 60% of maximal aerobic capacity. A minimum of 5 d between crossover phases was enforced.	Drug exposure: DRI (40 mg methylphenidate), NRI (8 mg reboxetine), and placebo	DA reuptake inhibition and norepinephrine reuptake inhibition prevented fatigue-related decrements in the peak velocity of prosaccades.
Rosso et al. [44]	Cohort, 1635 sedentary adults at risk for disability, 65.9% women, aged 78 ± 5.2, USA.	PA was calculated from accelerometry (min/d) at baseline, 6, 12, and 24 months. PA versus health education for an average of 2.6 years. PA intervention consisted of walking (goal of 150 min/week), strength, flexibility, and balance training.	No drug exposure: Single nucleotide polymorphisms of dopamine-related genes (dopamine receptor (DR) D1, DRD2, DRD3, and catechol-O-methyltransferase)	Higher dopamine signalling may support changes in PA during an intervention.

Abbreviations: DA, dopamine; VE, breath-by-breath ventilation; VCO_2_, CO_2_ production; VO_2_, O_2_ consumption; HA1, HA3, and HA5, days 1, 3 and 5 respectively at high altitude; ACTH, adrenocorticotropic hormone; CPET, cardiopulmonary exercise testing; DR, dopamine receptor; PA, physical activity; SNPs, single nucleotide polymorphisms; TT, trial time; TTE, time to exhaustion; MVPA, minutes of moderate-to-vigorous physical activity.
